# Differential Speed and Accuracy Trade‐Off in Working Memory Retrieval and Bilateral Precuneus Between Older Men and Women

**DOI:** 10.1002/brb3.70912

**Published:** 2025-09-25

**Authors:** Darlingtina K. Esiaka, Stephanie Strothkamp, Aghayeeabianeh Banafsheh, Lucas Broster, David K. Powell, Gregory Jicha, Yang Jiang

**Affiliations:** ^1^ Department of Behavioral Science University of Kentucky College of Medicine Lexington Kentucky USA; ^2^ Center For Health, Engagement and Transformation University of Kentucky College of Medicine Lexington Kentucky USA; ^3^ Sanders‐Brown Center on Aging University of Kentucky College of Medicine Lexington Kentucky USA; ^4^ MRISC Universityof Kentucky College of Medicine Lexington Kentucky USA

**Keywords:** cognitive decline, precuneus volumes, sex differences, working memory retrieval

## Abstract

**Background:**

Despite various hypotheses, including differences in longevity, hormones, genetics, and neuroanatomy, the reasons for the higher prevalence of Alzheimer's disease in older women compared to men remain unclear. Emerging evidence suggests that the precuneus, a key region of the default mode network, is linked to internally focused processes like memory retrieval. We examined the relationship between precuneus volumes and task performance (accuracy and reaction time) during working memory retrievals in cognitively normal older adults.

**Method:**

A cohort of older participants (*N* = 45; 25 women; *M*_age_ = 77) from the University of Kentucky Alzheimer's Disease Research Center (UK‐ADRC) performed the modified delayed match‐to‐sample working memory task while undergoing functional magnetic resonance imaging in a 3T Siemens scanner.

**Result:**

We found the accuracy of working memory retrieval correlates more frequently with the left precuneus in women (*r* = 0.54; *p* < 0.05) than in men. Similarly, volumes in the left precuneus displayed a significant negative correlation with reaction time in response to memory target (*r* = −0.426; *p* = 0.038) and memory distractor (*r* = −0.549; *p* < 0.01) in women. There is a sex difference in the accuracy and speed trade‐off. While men were faster in reaction time, women were better at the accuracy of the memory task. We found no sex differences in the percentage of fMRI signal change across reaction time in response to the memory target for men (*R* ^2^ linear = 0.011) and women (*R* ^2^ linear = 0.003). Particularly noteworthy was the consistent association in women, where neurocognitive measures (trail A, *r* = −0.50; *p* = 0.010; trail B, *r* = −0.06; *p* = 0.002) reliably correlated with volumes in the left precuneus—a relationship not observed in men.

**Discussion:**

Our findings suggest that the left precuneus volume is associated with processing speed and accuracy of working memory performance, especially in women. Given that the left precuneus plays a key role in supporting various aspects of cognition, including memory retrieval, our findings point to the potential of reaction time serving as a surrogate marker for functional MRI in predicting cognitive decline, particularly in older women.

## Graphical abstract

This study examined the relationship between precuneus volumes and task performance (accuracy and reaction time) during working memory retrievals in cognitively normal older adults. The result shows that left precuneus volume is associated with memory accuracy and processing speed in older women.

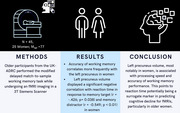

## 1 Introduction

Alzheimer's disease (AD) is a progressive neurodegenerative disorder associated with impairments in various cognitive functions, including working memory (Kirova et al. 2015). AD disproportionately affects women (Ferretti et al. 2018), with approximately two‐thirds of those affected by AD being women (Mielke et al. 2014). While the precise cause of sex differences in AD remains unclear, numerous studies have associated various factors with the observed distinctions between men and women in AD.

The examination of sex differences in AD often contemplates the longevity theory, given that women generally outlive men, making age a significant risk factor for AD (Alzheimer's Association 2024). Nevertheless, conflicting evidence exists regarding whether age‐adjusted AD risk is higher in women. Some studies found no significant sex differences in AD incidence (Fiest et al. 2016; Neu et al. 2017), while others suggested higher rates in women (Altmann et al. 2014), especially after the age of 80 (Ruitenberg et al. 2001; Roberts et al. 2014). In addition, hormonal changes during menopause have been implicated in the elevated risk of AD in women. Research indicates that biomarker abnormalities associated with AD are most pronounced in postmenopausal women (Mosconi et al. 2017; Oveisgharan et al. 2018).

In addition, the apolipoprotein ε4 allele (APOE4) has been identified as a factor contributing to AD risk between sexes, with women carrying APOE4 experiencing faster cognitive decline (Altmann et al. 2014; Mosconi et al. 2017). Moreover, sex differences in AD manifestation extend to women exhibiting more severe neuropsychiatric symptoms, neuroinflammation, and cognitive impairments (Cui et al. 2023; Eikelboom et al. 2022; Laws et al. 2018). Significantly, structural brain differences and anatomical changes in mild cognitive impairment and AD have been observed, indicating distinct effects on men and women (Ardekani et al. 2016; Cavedo et al. 2018; Kim et al. 2015; Koran et al. 2017). These findings contribute to a deeper understanding of the complex nature of sex differences in AD, encompassing genetic, hormonal, neurocognitive, and pathophysiological factors.

A growing body of evidence points to the importance of regions outside the traditional focus of the medial temporal lobe, hippocampus, particularly the precuneus, in predicting AD progression. The precuneus, situated in the posterior medial parietal cortex, plays a crucial role in visual‐spatial image processing and episodic memory retrieval (Dadario and Sughrue 2023; Dordevic et al. 2022; Eskildsen et al. 2015). Studies suggest that decreased precuneus volumes may serve as an indicator of AD pathology, independent of medial temporal lobe involvement (Billette et al. 2022; Casula et al. 2023; Haussmann et al. 2017; Mahady et al. 2021; Richter et al. 2022). Billette et al. (2022) used a novelty‐related fMRI activity in the precuneus to show that increased precuneus activity might be an early indicator of memory impairment. A 2017 study found that patients with Amnestic mild cognitive impairment (aMCI)—a clinical syndrome characterized by isolated memory deficits beyond normal age‐related changes, while daily functioning remains largely preserved (Gauthier et al. 2006)—demonstrated cortical thinning in the precuneus (Haussmann et al. 2017).

Sex‐specific differences in precuneus connectivity further contribute to the complexity of AD research (Williamson et al. 2022; Zhang and Chiang‐shan 2012), highlighting the need to consider both structural and functional aspects. Zhang and Chiang‐shan (2012) observed sex differences in precuneus connectivity, with men exhibiting greater connectivity with the dorsal precuneus in the cuneus and medial thalamus, while women showed increased connectivity with the ventral precuneus in the hippocampus/parahippocampus, middle/anterior cingulate gyrus, and middle occipital gyrus compared to men. Similarly, Williamson et al. (2022) examined sex differences in the hippocampal connectivity to different areas of the brain and found that the hippocampal connection to the precuneus cortex was significantly stronger in men than in women. Overall, despite various hypotheses, including differences in longevity, hormonal changes, genetics, neuroanatomy, and pathophysiology, the precise reasons for the higher prevalence of AD in older women compared to men remain unknown. Understanding these differences may offer valuable insights into developing targeted interventions for cognitive health.

In recent years, working memory—the cognitive system responsible for temporarily holding and manipulating information with or without emotional content has emerged as a crucial domain in understanding AD risks (Borhani et al. 2021; Zheng et al. 2023). There is growing evidence that deficits in working memory may serve as an early indicator or a contributing factor to the development of cognitive impairments (Kjærstad et al. 2023). Also, sex differences in working memory performance have been a subject of considerable research interest, with findings suggesting nuanced patterns. Some studies indicate that women exhibit advantages in verbal working memory tasks (Goldstein et al. 2005; Hirnstein et al. 2023; Voyer et al. 2021). Others find that men show advantages in spatial working memory tasks (Chen et al. 2020). Mixed findings on sex differences have also been reported regarding the accuracy, reaction times (RTs), and false alarm rates during memory performance. Subtle variations in these parameters have been observed between men and women (Chang and Moscovitch 2022; Samson et al. 2023), with women outperforming men in immediate and delayed recall (Sundermann et al. 2016). However, Vaughan and Birney (2023) emphasize the importance of considering individual variability and the specific nature of the memory tasks employed. There remains a need for a more nuanced and multifaceted understanding of sex differences in working memory.

Determining if and where sex differences exist in AD is critical for the direction of future targeted diagnostics and therapeutic interventions. Evidence from aMCI populations has provided critical insights into early AD‐related neural changes, including alterations in the medial temporal lobe and posterior cortical regions. However, studying cognitively unimpaired older adults is essential for identifying even earlier, preclinical brain changes that precede measurable cognitive decline. The current study aims to explore potential sex differences in memory performance and precuneus brain volume among cognitively normal older adults. Specifically, we examined the volume of the precuneus region involved in memory and compared participants’ RT and accuracy performance on a memory task. We hypothesized that (H1) there will be differential effects of precuneus volumes on network‐associated working memory performance in cognitively normal older men versus women, such that precuneus volume will be (H1a) negatively associated with RT and (H1b) positively associated with accuracy in response to a performance task in women. As a corollary of H1, we hypothesize that (H2) greater precuneus volume will be associated with better performance on the Trail Making Task—a neuropsychological test of visual attention and task switching—reflecting its role in visuospatial processing, working memory, and executive function. By investigating the relationship between precuneus volume and memory tasks, we aim to shed light on how deficits or alterations in working memory may serve as potential predictors or indicators of cognitive decline, providing valuable insights for early detection of cognitive decline.

## 2 Materials and Methods

### 2.1 Participants

Overall, fifty‐two (52) cognitively normal older adults (*m*_age_  =  76.7 years; 27 women) were recruited from the University of Kentucky Alzheimer's Disease Center cohort for the study participation. All of the participants were racialized as non‐Hispanic White Americans. Data from seven participants were removed from the analysis based on the exclusion criteria—the presence of dementia, double participation in the study, and missing data on neuropsychological tasks. One participant had dementia, while six participants completed the test two times. The second records of the six participants, which also had more missing data in their behavioral task, were removed. A total of 45 participants were included (*M*_age _=  77.05, *SD*_age_ = 7.48; 25 women) in the present study. All research activities were approved by the University of Kentucky Institutional Review Board, and all participants provided written informed consent.

### 2.2 Measures

#### 2.2.1 Bluegrass Short‐Term Memory Task

**FIGURE 1:**
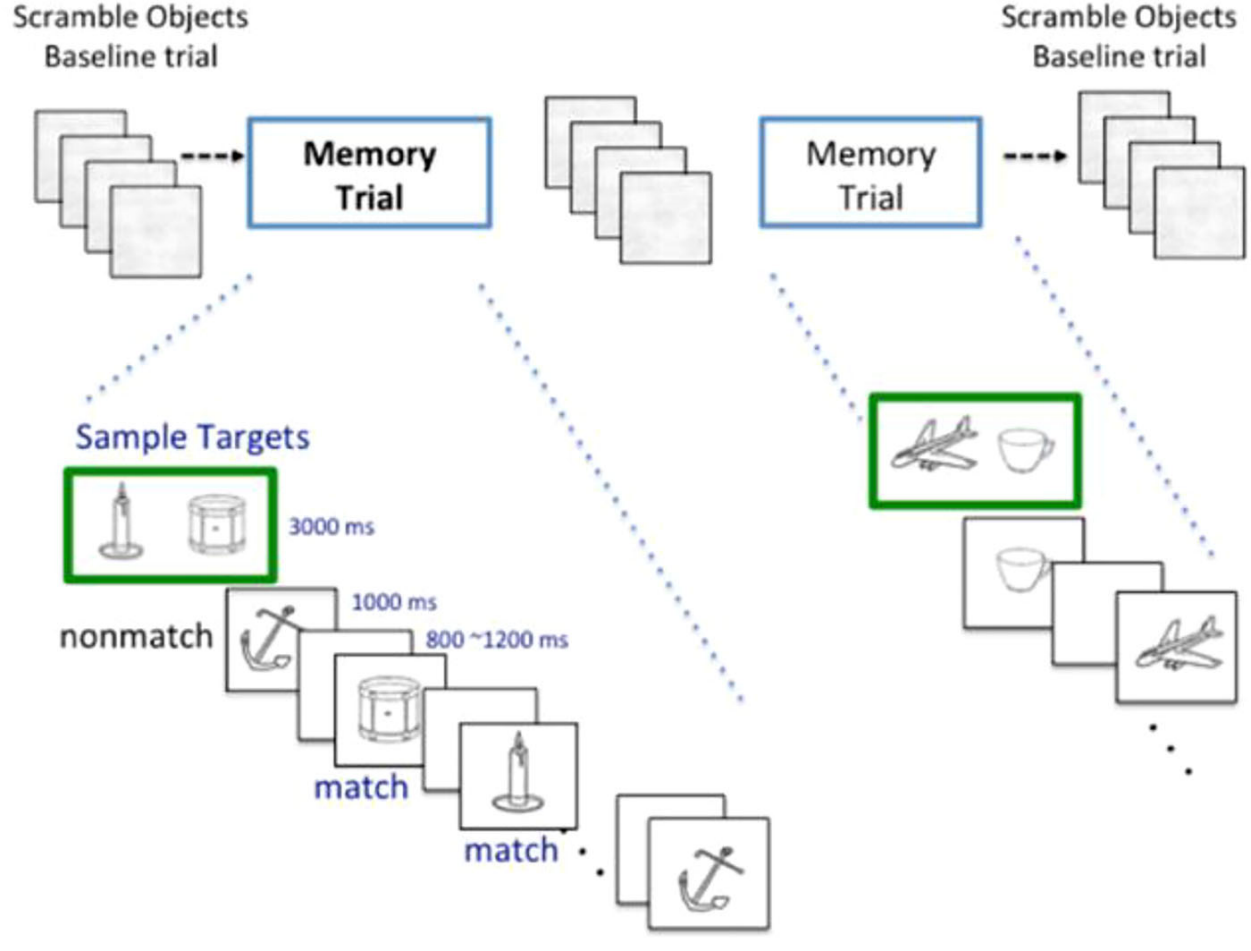
The Bluegrass short‐term Memory task (BeST) is described in Jiang et al. (2016). In this task, participants memorize two sample images and then indicate whether each image presented thereafter matches either sample target image.

The Bluegrass Short‐Term Memory Task (BeST) is an adapted version of a previous fMRI delayed match‐to‐sample memory task (Jiang et al. 2000) and previously described (Jiang et al. 2016). The task uses a 10‐min run time in which two pictures/images are encoded in each trial. Pictures of common images (e.g., candle, hammer, etc.) are used as the targets and distractors (i.e., match and nonmatch). In the BeST 10‐min older‐adult friendly version, two sample pictures were encoded for each given trial. This reduces scanning time for older adults and increases the number of matches with a balanced number of non‐matches. Images are scrambled between trials to ensure the baseline of activity is reached. A representation of the task is shown in Figure 1.

Memory performance was characterized by accuracy and RT. We measured accuracy by the percent/ratio of correct answers and false alarms. A false alarm is the action of identifying a non‐match visual object as a match target held in memory in a delayed match‐to‐sample paradigm. Participants are shown two target items to remember at the beginning of a trial and asked to determine whether or not other items seen in the trial are a match or non‐match (e.g., thinking a car in a parking lot is yours when it is not). Participants switched hands, indicating memory targets (hereafter referred to as target images) and non‐targets (hereafter referred to as distractor images) between blocks. Thus, the RTs of memory retrieval of targets and distractors are balanced within a subject. In this study, we performed two trials of the BeST Memory Task for each participant, looking at the performance for both targets and distractors. False alarm rate (FA_Rate), which increases with cognitive aging, was also measured. Accuracy and RTs were measured for the first and second‐trial match and non‐match targets. False alarm number (FA_N), FA_Rate, and false alarm RT (FA_RT) were also measured.

#### 2.2.2 Trail Making Test

The trail making test (TMT; Bowie and Harvey 2006) is a neuropsychological test that provides information on visual search, scanning, speed of processing, mental flexibility, and executive functions. The TMT consists of two parts—TMT‐A requires an individual to draw lines sequentially connecting 25 encircled numbers distributed on a sheet of paper. Task requirements are similar for TMT‐B, except the person must alternate between numbers and letters (e.g., 1, A, 2, B, 3, C, etc.). Participants were instructed to complete each part of the TMT as quickly and accurately as possible. The time to complete each part was recorded. Each part's score represents the time required to complete the task.

#### 2.2.3 Neuroimaging Procedures

MRI images were obtained with a 3‐T Siemens Trio Scanner at the Magnetic Resonance Imaging and Spectroscopy Center (MRISC) of the University of Kentucky using a 32‐channel head coil. High‐resolution anatomic images (20 min 3D MPRAGE) were acquired using a rapid gradient echo acquisition sequence (acquisition matrix 256 × 256 × 176, isotropic 1 mm voxels, field of view 256 mm, repetition time 2530 ms, echo time 2.26 ms). The resting‐state functional MRI (fMRI) was acquired with the following parameters: TR = 2 s; TE = 30 ms; flip angle = 76°; 39 axial slices; FOV = 224 × 224 mm; slice thickness = 3.5 mm; matrix = 64 × 64; bandwidth = 2056 Hz/Px. A total of 200 volumes of resting‐state data were acquired. The task‐based fMRI scan was acquired using a T2*‐weighted gradient echo EPI sequence, TR = 2 s; E = 30 ms; flip angle = 81°; 39 axial slices; FOV = 224 × 224 mm; slice thickness = 3.5 mm; matrix = 64 × 64; bandwidth = 2056 Hz/Px. With MRI and as part of a larger study, we analyzed the volume of multiple regions of interest, including the hippocampus, amygdala, dorsolateral prefrontal cortex (DLPFC), frontal eye field (FEF), precuneus, inferior parietal lobe (IPL), insula, and the fusiform.  The volumes of each region of interest were normalized to the total intracranial volume. CSF samples of Aß_42 _and p‐Tau_181 _were taken from all participants as described in the Jiang et al. (2016) study. In this study, we present findings on precuneus volume.

### 2.3 Data Analysis

Descriptive statistics were performed to calculate participants’ demographic and clinical characteristics. Given the modest sample size (*N* = 45), we conducted sex‐stratified Pearson correlation analyses, using pairwise deletion, to explore the associations between brain measures and cognitive outcomes within each sex group. This approach was selected in lieu of a formal interaction analysis (e.g., multiple regression with interaction terms), as such models require larger sample sizes to yield reliable estimates and sufficient statistical power. While Pearson correlations do not formally test interaction effects, comparing correlation coefficients across sex groups provides preliminary insight into potential sex‐specific patterns in brain–cognition relationships. This exploratory method offers a statistically conservative approach appropriate for the current sample size. In addition, Pearson *r* correlation analyses were performed on the neuropsychological and fMRI data with age and education controlled to account for potential confounding variables. We determined statistical significance with the probability of a Type I error set at *p* ≤ 0.05 and conducted all statistical analyses using SPSS version 30.0 (SPSS Inc., Chicago, IL). No correction for multiple comparisons was applied; therefore, results should be interpreted with caution regarding potential inflation of Type I error.

## 3 Results

### 3.1 Participant Demographics

Participants’ demographic and clinical characteristics are summarized in Table 1. The mean participant age was 77.05 (±16.54), with a range of 59–93 years. The mean number of years of formal education was 16.54 (±2.46), and 55.6% of the subjects were women. Age (*p* = 0.870) and level of education (*p* = 0.412) did not differ significantly between men and women. In addition, there were no significant differences in mean A‐Beta42 (*M* = 265.88, SD = 81.26) and mean p‐Tau181P (*M* = 23.47, SD = 7.68) between men and women.

**TABLE 1:** Demographic and clinical characteristics (*N* = 45).

Variable	All participants	Men	Women	*p* value
	M (SD)	*n* = 20 M (SD)	*n* = 25 M (SD)	
Age	77.05 (7.48)	76.83 (8.08)	77.24 (7.11)	0.870
Education	16.54 (2.46)	16.89 (2.30)	16.24 (2.61)	0.413
Trail A	35.68 (15.20)	33.28 (12.08)	37.85 (17.57)	0.353
Trail B	90.76 (54.19)	85.67 (60.11)	95.35 (49.39)	0.593
A‐Beta42	265.88 (81.26)	264.26 (81.04)	267.17 (83.15)	0.909
p‐Tau181P	23.47 (7.68)	23.42 (6.86)	23.50 (8.42)	0.973

### 3.2 Sex Differences in Memory Task Performances

**FIGURE 2:**
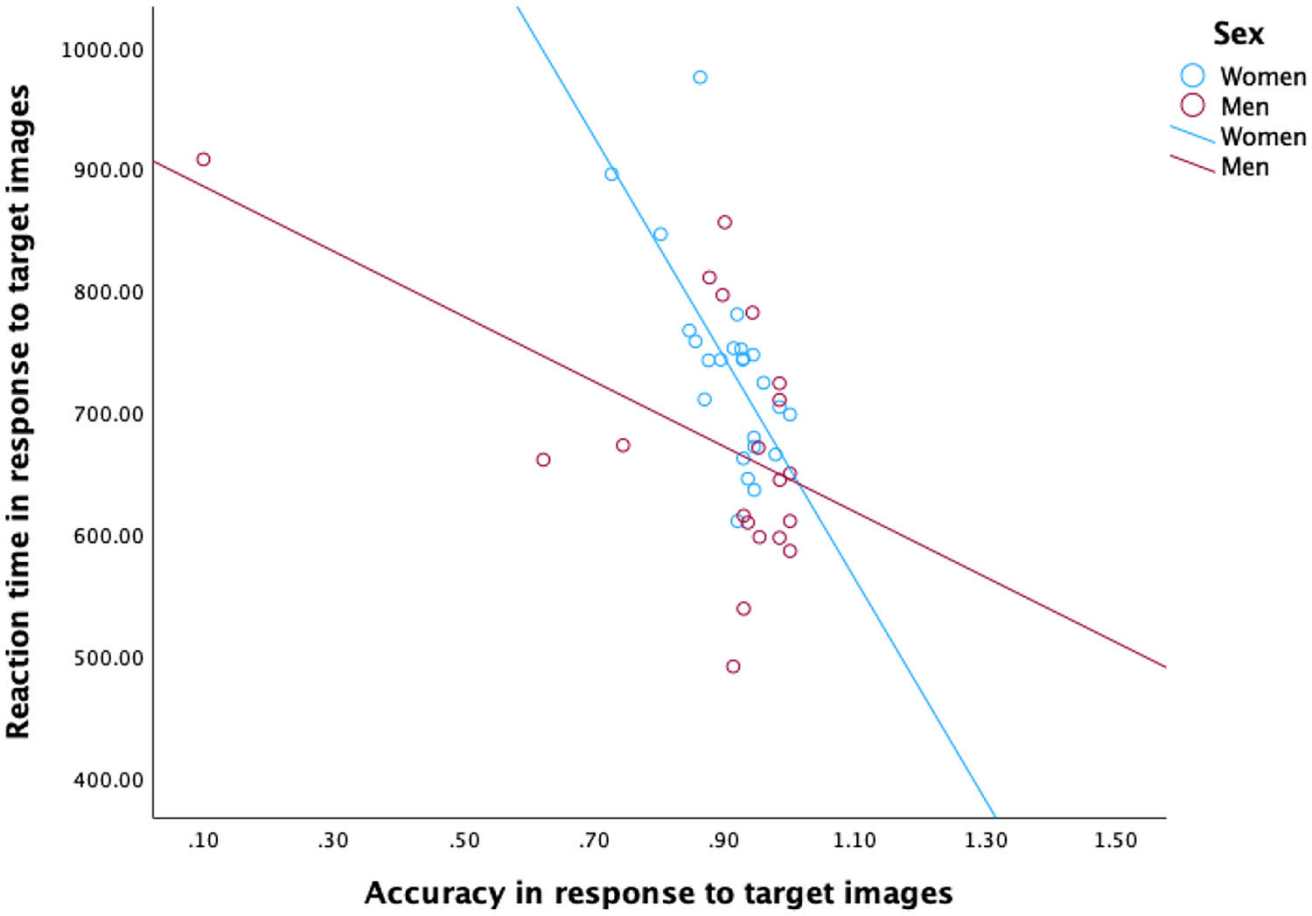
Sex differences in accuracy (circle) and interaction trade‐off of reaction times in response to memory target during working memory retrieval.

Figure 2 illustrates the relationship between accuracy in identifying target images and RT, stratified by sex. Higher accuracy was associated with shorter RTs. Both groups demonstrated the same directional trend, consistent with a speed‐accuracy relationship in memory task performance. However, the negative association appeared steeper in women than in men, suggesting that accuracy was more strongly linked to reaction speed in women. In line with the anticipated patterns, men and women differed in their RT to distractor images (*p* = 0.023), their RT to correct responses (*p* = 0.002), and their false alarm reaction rate (*p* = 0.044). Women responded more slowly to distractor images than men (H1a) and produced correct responses (H1b). Also, women were slower in reacting to false alarms compared to men. Thus, showing higher accuracy in memory tasks than men. See Table 2 for additional information.

**TABLE 2:** Descriptives result of memory performances.

Variables	All participants	Men	Women	*p* value
	M (SD)	*n* = 20 M (SD)	*n* = 25 M (SD)	
RT1	708.76 (98.09)	676.51 (107.96)	735.64 (81.86)	0.052
RT2	745.27 (93.21)	709.40 (104.66)	775.15 (71.73)	0.023
CR_RT	724.70 (85.69)	679.46 (83.70)	763.47 (67.64)	0.002
FA_RT	728.85 (193.67)	656.83 (165.76)	792.39 (198.68)	0.044
CR_Rate	0.91 (0.11)	0.92 (0.12)	0.91 (0.11)	0.788
AC1	0.90 (0.14)	0.88 (0.21)	0.91 (0.06)	0.530
AC2	0.95 (0.08)	0.94 (0.11)	0.96 (0.04)	0.424
CR_N	88.49 (13.23)	90.11 (11.37)	87.10 (14.77)	0.476
FA_N	4.62 (3.56)	4.40 (3.25)	4.82 (3.91)	0.740

Abbreviations: AC1 = Accuracy in response to target images; AC2 = Accuracy in response to distractor images; CR_N = correct response number; CR_Rate = correct response rate; CR_RT = Reaction times of correct responses; FA_N = false alarm number; FA_RT = false alarm reaction time; RT1 = Reaction time in response to target images; RT2 = Reaction time in response to distractor images.

### 3.3 Precuneus Deactivation Correlates With Faster Reaction Time

**FIGURE 3:**
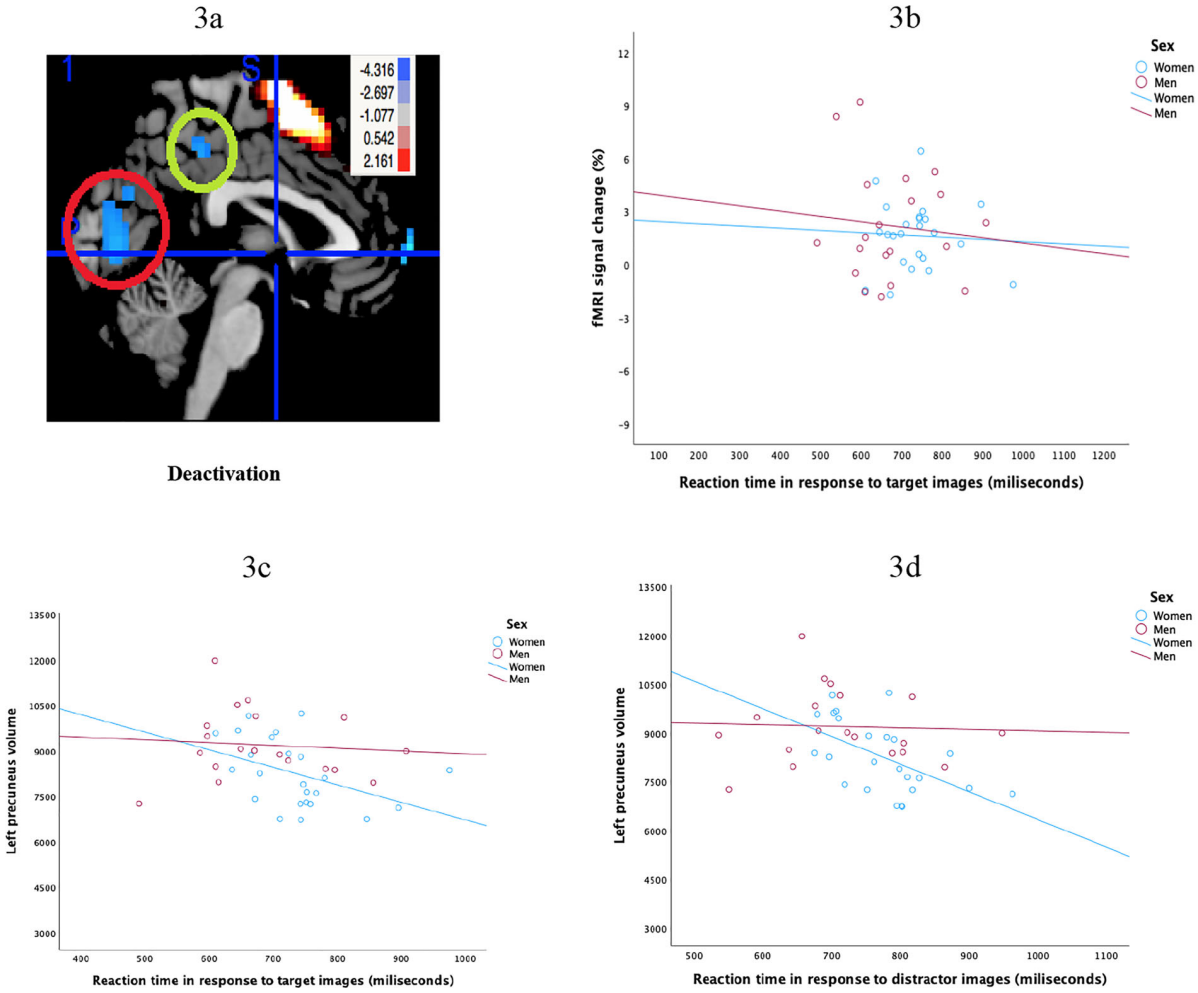
(a) shows the deactivation of fMRI‐BOLD signals in the precuneus. (b) shows the percentage of fMRI signal change across reaction time in response to memory target for men (*R* ^2^ linear = 0.011) and women (*R* ^2^ linear = 0.003). No sex difference. (c) shows the relationship between left precuneus volume and reaction time in response to memory target in men (*R* ^2^ linear = 0.007) and women (*R* ^2^ linear = 0.182), while (d) shows the relationship between left precuneus volume and reaction time in response to distractor images in men (*R* ^2^ linear = 0.002) and women (*R* ^2^ linear = 0.302).

We examined the relationship between memory tasks and resting‐state functional brain connectivity. Figure 3a shows the deactivation of the precuneus in milliseconds, while Figure 3b illustrates the relationship between fMRI signal change during precuneus deactivation and RT in response to target images, stratified by sex. A negative association was observed in both women and men, such that greater precuneus deactivation was associated with faster RTs. Visual inspection suggests that the association may be slightly stronger in women. These findings suggest a general pattern in which more pronounced suppression of precuneus activity relates to more efficient cognitive responses during memory tasks.

### 3.4 Precuneus Volumes Display Negative Correlation With RT in Women but Not in Men

Figures 3c,d shows the relationship between left precuneus volume and performance on the working memory task. The results (see Table 3) show that in women, volumes in the left precuneus displayed a significant negative correlation with RT in response to target images (*r* = −0.426; *p* < 0.05) and distractor images (*r* = −0.549; *p* < 0.01). As volumes in the left precuneus decreased, RT in response to both target and distractor images increased. There was no significant relationship between left precuneus volumes and RT in response to target or distractor images in men. The results also show volumes in the left precuneus displayed a significant negative correlation with RT to correct responses in women (*r* = −0.580; *p* < 0.01). As the volumes in the left precuneus increased, RT to correct responses decreased.

**TABLE 3:** Correlations between left precuneus volumes, and reaction time, accuracy of BeST task and Trailmaking task in men and women.

Left precuneus	*r*_all subjects_	*p* value	*r*_men_	*p* value	*r*_women_	*p* value
Reaction time in response to target images	−0.330	0.031	−0.085	0.729	−0.426	0.038
Reaction time in response to distractor images	−0.367	0.016	−0.044	0.859	−0.549	0.005
Reaction times of correct responses	−0.378	0.019	0.062	0.814	−0.580	0.006
Reaction times of false alarms	−0.376	0.037	0.008	0.979	−0.488	0.047
Accuracy in response to target images	0.020	0.901	−0.098	0.689	0.559	0.005
Accuracy in response to distractor images	0.031	0.846	−0.012	0.962	0.401	0.052
Number of correct responses	0.200	0.288	−0.030	0.909	0.310	0.171
TRAIL A	−0.551	0.001	−0.515	0.034	−0.563	0.010
TRAIL B	−0.340	0.040	−0.047	0.857	−0.658	0.002

**FIGURE 4:**
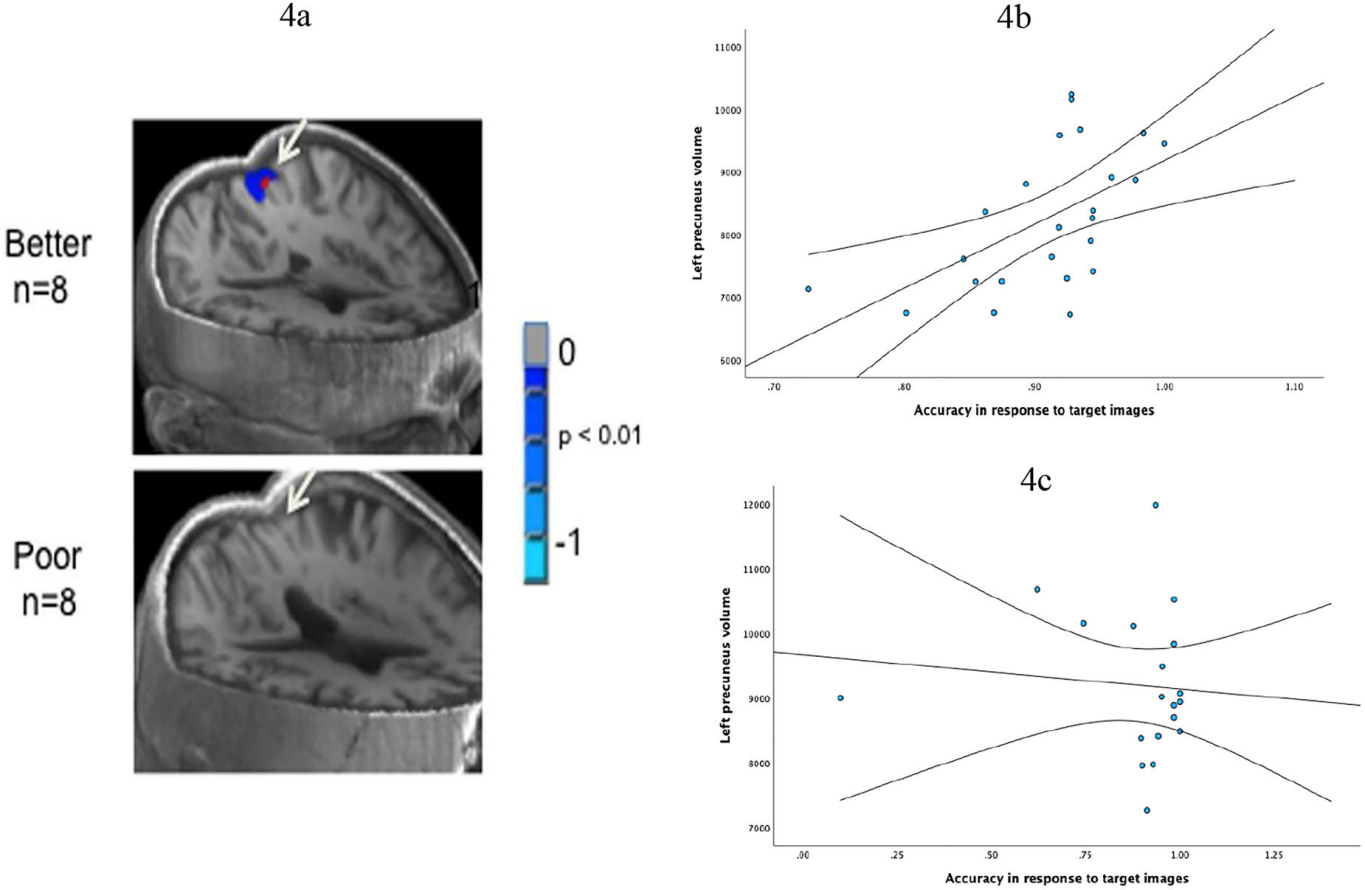
(a) shows the top eight versus bottom eight performers in a delayed match‐to‐sample working memory task. Better performers on the working memory task showed deactivation of posterior precuneus/cingulate (top) during the working memory task, whereas poor performers failed to show the deactivation pattern (bottom). (b) Shows the correlation between left precuneus volume and accuracy in response to target images in women. (c) Shows there is no correlation between left precuneus volume and accuracy in response to target images in men.

There was no significant relationship between left precuneus volumes and RT to correct responses in men. In addition, the results show that volumes in the left precuneus displayed a significant negative correlation with RT to false alarms (*r* = −0.488; *p* < 0.05) in women. As volumes in the left precuneus increased, RT to false alarms decreased. There was no significant relationship between left precuneus volumes and RT to correct responses in men. Figure 4a shows the deactivation patterns of the posterior precuneus in the participants during the working memory task. Further, we found that volumes in the left precuneus displayed significant positive correlation with accuracy in response to target images (*r* = 0.559; *p* < 0.01; Figure 4b) and distractor images (*r* = 0.401; *p* = 0.052) in women. As the volumes in the left precuneus increased, accuracy in response to both target and distractor images also increased. There was no significant relationship between left precuneus volumes and accuracy in response to target (Figure 4c) or distractor images in men. Also, volumes in the left precuneus displayed no significant correlation with the total number of correct responses in both men and women. See Table 3 for detailed information.

As an exploratory analysis, we examined the relationship between precuneus volume and Trailmaking—a neuropsychological test of visual attention and task switching (H2a). Table 3 shows that volumes in the left precuneus displayed significant negative correlation with both Trail Making task A (*r* = −0.563; *p* = 0.010) and B (*r* = −0.658; *p* < 0.01) in women. The correlation was stronger with Trails A than Trails B, an effect that may be driven by processing speed rather than working memory/executive function. Thus, confirming our general findings relating to RT. As the volumes in the left precuneus increased, the time needed to complete trailmaking tasks decreased. Also, volumes in the left precuneus displayed a significant negative correlation with the trail making task A in men (*r* = −0.515; *p* < 0.05). There was no significant relationship between left precuneus volumes and time to complete trail making task B in men.

## 4 Discussion

As the number of people at risk of developing AD increases, the characterization of sex differences in AD risk is important for the direction of future research, precision diagnoses, and therapeutic interventions (Mielke 2020; Nebel et al. 2018). We aimed to examine potential sex differences in the precuneus volume and compared the participants' performance on memory and neuropsychological tasks. By studying sex differences in healthy individuals, researchers may gain insights into how these differences contribute to AD risk, progression, and treatment response while addressing the complex interplay between biological factors and memory‐related outcomes.

We hypothesized that sex differences would have differential effects on brain volumes and memory task performance in cognitively normal older adults. We found that in women, measures of memory performance correlated consistently with volume in the left precuneus. While men were faster in RT, women were better in accuracy. These relationships were consistent and in expected directions given units of measure (e.g., negative correlation between left precuneus volume and trail‐making task score, but positive correlation between left precuneus volume and accuracy). Our study supports prior findings showing sex‐specific patterns in brain structure changes that may influence the onset and development of Alzheimer's pathology (Medeiros and Silva 2019; Samson et al. 2023; Williamson et al. 2022). By identifying that women exhibit slower RT but better accuracy compared to men in a memory task, this study contributes to a deeper understanding of the neural mechanisms underlying cognitive processes and highlights the need for further exploration of sex‐related differences in cognition.

Atrophy of brain structures has been continuously recognized as an early and key feature of AD (Jiang et al. 2016; Richter et al. 2022; Rusinek et al. 2004; Williamson et al. 2022). Since the precuneus plays a critical role in episodic memory retrieval, early changes in the precuneus can signal memory impairment—a precursor for AD. Studies show that AD pathology tends to follow a characteristic pattern of progression, starting in the medial temporal lobe and spreading to other brain regions over time (Rusinek et al. 2004). This temporal progression aligns with the clinical observation that memory deficits are often among the earliest symptoms of the disease (Aël Chetelat and Baron 2003). Similarly, autopsy studies of individuals with AD consistently reveal significant atrophy in brain structures (de Flores et al. 2020; Nedelska et al. 2015; Whitwell et al. 2008). In line with the current study, advanced neuroimaging techniques, such as fMRI, allow us to visualize structural changes in the brain and the associated cognitive and neurological conditions. Future direction would include characterizing participants according to A‐beta and pTau profiles to assess whether the observed sex differences in fMRI biomarkers persist across these biomarker‐defined subgroups.

Our findings point to the potential of RT serving as a surrogate marker for fMRI in predicting cognitive decline, particularly when considering sex differences. Research has shown that slower RTs are associated with cognitive impairment and decline, as they may reflect deficits in processing speed, attention, and executive function, which are early indicators of cognitive decline (Haynes et al. 2017; Velichkovsky et al. 2020). We showed that women tend to have slower RTs compared to men, but they may compensate with better accuracy in memory tasks. Therefore, monitoring changes in RT, especially in conjunction with fMRI measures of brain function, could provide valuable insights into sex‐specific patterns of cognitive decline. By integrating RT as a surrogate marker with fMRI data, researchers can better understand the underlying neural mechanisms of cognitive decline and potentially identify sex‐specific biomarkers for early detection and targeted interventions.

In addition to adding to the evidence that there are sex differences in AD risk factors, our findings have the potential to significantly inform targeted interventions aimed at preserving cognitive function in affected individuals. One strength of the present study is that many memory performance metrics were collected, including RT, accuracy, and false alarms. Other neuropsychological test of executive function and processing speed (i.e., trail making tasks) was also collected. This variety of data allowed for the analysis of multiple aspects of memory. Although many studies have reported developmental differences in the parietal lobe between boys and girls, very little is known about brain and cognitive aging related to the brain structure. The current study is unique in terms of sex‐specific working memory performances in bilateral precuneus function in the aging population.

Some limitations are worth noting. The present study has a relatively small sample size. A larger sample size may yield findings that are different from those in the current study. The sample consists entirely of non‐Hispanic Whites; therefore, our findings are not generalizable to racially diverse older Americans. In addition, while our findings focus on the precuneus as an important hub in the default mode network (DMN), we recognize that this brain region is also involved in other brain networks, and we did not evaluate the connectivity of the precuneus with other areas within the DMN or other networks. In the future, we hope to focus our measurements on the DMN to further characterize its relationship with memory performance in cognitively normal older adults and how this relationship may differ between men and women. Furthermore, we hope to characterize how this relationship changes over time. The present study examined only cognitively normal older adults, but future studies should investigate these differences in adults with clinical AD. Finally, due to the limited sample size, we were underpowered to conduct formal interaction analyses to test for sex differences in brain–cognition associations. However, our approach provides preliminary insights that may guide future studies with larger samples and sufficient power for interaction modeling.

## 5 Conclusion

The current study shows that by studying sex differences in brain volumes, researchers may gain insights into how sex differences contribute to memory impairment and AD risk. Identifying sex‐specific factors that influence memory can guide the development of targeted preventive measures and interventions. This is particularly relevant as researchers explore ways to promote cognitive health and resilience against age‐related memory decline. Since women tend to have a higher prevalence of certain neurodegenerative disorders, understanding sex differences in memory‐related brain regions can inform policies, healthcare strategies, and resources for managing and preventing memory‐related disorders.

## Author Contributions

**Darlingtina Esiaka**: writing – original draft, formal analysis, visualization, writing – review – editing. **Stephanie Strothkamp**: investigation, writing – original draft, formal analysis, data curation. **Aghayeeabianeh Banafsheh**: writing – original draft, formal analysis, data curation. **Lucas Broster**: formal analysis, data curation, software. **David Powell**: methodology, data curation, funding acquisition. **Gregory Jicha**: data curation, resources, funding acquisition. **Yang Jiang**: conceptualization, writing – review & editing, resources, data curation, formal analysis, funding acquisition.

## Conflicts of Interest

The authors declare no conflicts of interest.

## Peer Review

The peer review history for this article is available at https://publons.com/publon/10.1002/brb3.70912.

## Data Availability

Data is available via the University of Kentucky Sanders Brown Center on Aging. https://medicine.uky.edu/centers/sbcoa/data‐sample‐request</meta‐value>.
